# Strain Echocardiography in Acute Cardiovascular Diseases

**DOI:** 10.5811/westjem.2015.12.28521

**Published:** 2016-01-12

**Authors:** Mark Favot, Cheryl Courage, Robert Ehrman, Lyudmila Khait, Phillip Levy

**Affiliations:** Wayne State University School of Medicine, Department of Emergency Medicine, Detroit, Michigan

## Abstract

Echocardiography has become a critical tool in the evaluation of patients presenting to the emergency department (ED) with acute cardiovascular diseases and undifferentiated cardiopulmonary symptoms. New technological advances allow clinicians to accurately measure left ventricular (LV) strain, a superior marker of LV systolic function compared to traditional measures such as ejection fraction, but most emergency physicians (EPs) are unfamiliar with this method of echocardiographic assessment.

This article discusses the application of LV longitudinal strain in the ED and reviews how it has been used in various disease states including acute heart failure, acute coronary syndromes (ACS) and pulmonary embolism.

It is important for EPs to understand the utility of technological and software advances in ultrasound and how new methods can build on traditional two-dimensional and Doppler techniques of standard echocardiography. The next step in competency development for EP-performed focused echocardiography is to adopt novel approaches such as strain using speckle-tracking software in the management of patients with acute cardiovascular disease. With the advent of speckle tracking, strain image acquisition and interpretation has become semi-automated making it something that could be routinely added to the sonographic evaluation of patients presenting to the ED with cardiovascular disease. Once strain imaging is adopted by skilled EPs, focused echocardiography can be expanded and more direct, phenotype-driven care may be achievable for ED patients with a variety of conditions including heart failure, ACS and shock.

## BACKGROUND

According to data from the Centers for Disease Control, cardiovascular disease remains the leading cause of death in the United States^1^ with more than 2,200 Americans succumbing to it each day.^2^ Many of the patients who ultimately die from heart disease present to the emergency department (ED) with medical issues that warrant acute intervention. While comprehensive, two-dimensional (2D) transthoracic echocardiography can provide critically important information to help guide treatment, it requires approximately 30–45 minutes to complete. The study requires a dedicated sonographer to perform and is interpreted offline by a cardiologist, which often fails to provide real-time actionable information at the point of care (POC). This time lag between performance and interpretation limits the utility of comprehensive echocardiography for the acutely ill patient where time-sensitive treatment decisions contribute directly to outcome. Thus, a more focused discerning approach to echocardiography that can be performed at the POC by adequately trained emergency physicians (EP) is often necessary.^3^,^4^

In 2010, the American College of EPs and the American Society of Echocardiography formed a joint committee and released a position paper entitled Focused Cardiac Ultrasound in the Emergent Setting, establishing standards for ED-based POC echocardiography.^3^ This consensus statement helped to solidify the core applications of POC echocardiography, which include the following: 1) assessment of the pericardium for pericardial effusion; 2) determination of global cardiac function; 3) evaluation of relative chamber size; 4) determination of volume status or preload; and 5) procedural guidance, specifically pericardiocentesis and insertion of a transvenous pacemaker. Components of focused echocardiography can be used in isolation for disease-specific indications such as acute heart failure (HF) or as a collective approach to conditions such as undifferentiated dyspnea or hypotension.^5^,^6^ In this five-year period since publication of the consensus statement, significant advances have been made in the field of echocardiography that can further expand the scope of EP-performed POC studies. Foremost among these is strain imaging using speckle tracking.

## STRAIN IMAGING

In the past decade, a new echocardiographic technique known as strain has been used to make more accurate assessments of the contractile state of the heart.^7^ Strain echocardiography has been used in a wide range of cardiovascular conditions including HF and acute coronary syndromes (ACS). Unlike conventional techniques of determining systolic function that rely on visual assessment of wall motion and changes in volume, strain echocardiography measures actual tissue deformation within the myocardium. Conceptually, strain can be thought of as a way to determine the movement between two points as if those two points are connected by a string. The physical definition of strain is the relative change in length of a material related to its original length. Mathematically, strain (ɛ) is calculated by subtracting the distance between two points at the start of a movement (L_0_) from the distance between them at the end of the movement (L_1_), and dividing it by the starting length (ɛ=ΔL/L_0_ where ΔL=L_1_–L_0_). Speckle tracking is a recent development in image processing that uses proprietary software to automatically determine the movements of these points, which appear sonographically as “speckles,” within the myocardium over time. Tracking the movements of these speckles throughout the cardiac cycle yields positive values when the two points are moving away from one another, as in diastole, and negative values when they are moving towards one another, as in systole. Speckle tracking obviates the need for Doppler-based imaging technologies to calculate strain, which have proved to be challenging due to their angle-dependency.^8^

**Figure 1:**
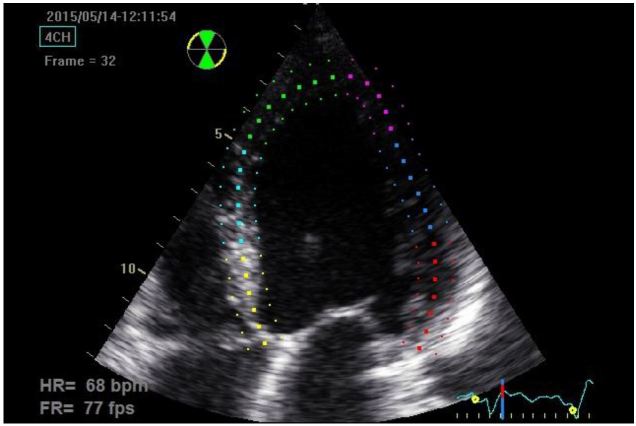
Apical 4-chamber image demonstrating semi-automated tracing of the endocardial border using two-dimensional speckle-tracking software. Depth is minimized excluding part of the left atrium in order to maximize frame rate.

**Figure 2:**
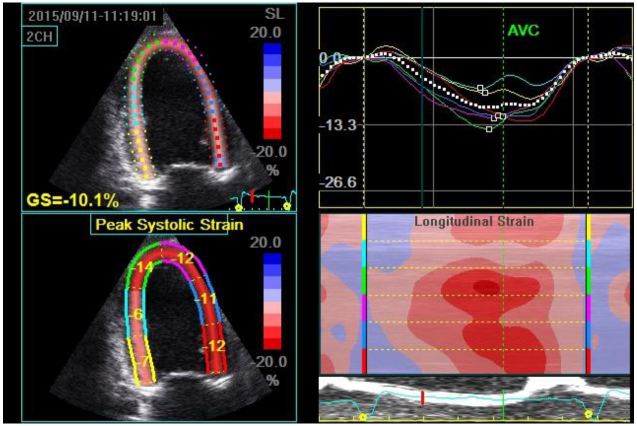
Quad display of an apical 2-chamber image demonstrating three different ways to depict left ventricular (LV) longitudinal strain. Images are taken from a patient with a history of heart failure with preserved ejection fraction. Top left image is a 2D depiction showing the color-coding for each LV segment and the global longitudinal strain in the 2-chamber plane (GS=−10.2%); bottom left image displays the strain for each of the 6 LV segments in the 2-chamber plane; bottom right shows the anatomical M-mode display for the 2-chamber plane with each LV segment color coded on the y-axis and the instantaneous strain being depicted using deeper red hues to represent more negative strain and deeper blue hues to represent more positive strain; top right displays strain (y-axis) plotted over time (x-axis) for each LV segment with a color-coded linear graphical display.

Strain can be measured in any of the longitudinal, circumferential or radial planes of the left ventricle (LV); however, the longitudinal orientation is likely the most useful, as impairment in this plane occurs earlier than in the other planes.^9^,^10^ If one is measuring longitudinal systolic strain, clips of the apical four-chamber (A4C), apical two-chamber and apical three-chamber views are acquired and saved. Once the exam is completed the physician-sonographer traces the endocardial border on the standard 2D images just as one would if they were using Simpson’s method to calculate the LV ejection fraction (LVEF). One tracing is made for each of the three clips during mid-systole (which is determined by the ultrasound system using a continuous electrocardiogram tracing). The software then delineates the endocardium, myocardium and epicardium and assigns speckle regions of interest within the myocardium of each segment (Figure 1). The LV is divided into 16–18 segments (depending on the ultrasound manufacturer’s software) with systolic strain calculated for each segment. The segmental data can be combined to give a global evaluation of myocardial strain called global longitudinal peak systolic strain (GLS), which in healthy subjects is between −18 to −22% in the longitudinal plane (Table).^11^ Once calculated, strain data can be depicted in numerous ways including the following: 1) on a simple linear graph with each segment of the LV being represented by a colored line where the x-axis is time (aortic valve closure is indicated by a green hashed vertical line) and the y-axis is strain; 2) on a curved anatomical M-mode display where each LV segment is represented on the y-axis using a different color block; and 3) using a “bullseye” map with the same red and blue color coding where the segments on the outermost ring represent basal segments, the middle ring the mid-wall segments and the innermost ring the apical segments (Figure 2). The various ways of depicting strain confer certain advantages for a given clinical scenario, such as the ease of analyzing for post-systolic shortening on the linear graph, which has been shown to be a poor prognostic marker in ischemic heart disease.^11^ Strain has potential clinical applicability for a variety of conditions relevant to EPs, some of which are outlined below.

### Acute Myocardial Infarction (MI)

LV strain on 2D echocardiography has been well studied in patients with ACS. For patients with ST-segment elevation MI (STEMI), LV GLS has been shown to be an important predictor of post-discharge adverse outcomes.^12^ Following non-ST segment elevation MI (NSTEMI), LV GLS can also help discriminate which patients will successfully recover LV function and which patients will develop adverse LV remodeling.^13^,^14^ Moreover, decreased GLS within 24 hours of revascularization for acute MI can reliably predict which patients are more likely to achieve a composite end-point of any of the following: all-cause mortality, hospitalization with re-infarction, HF, or stroke at six-month follow up.^15^

**Figure 3:**
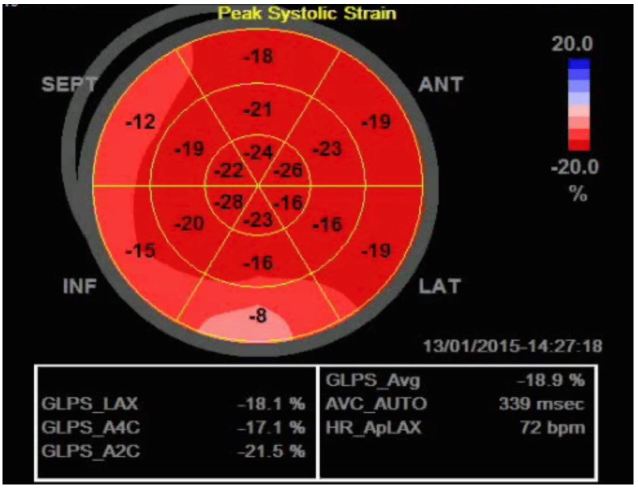
Bullseye map of left ventricular strain. Impaired longitudinal strain of the basal inferior wall is shown in a patient with an indeterminate troponin who would go on to develop a non-ST elevation myocardial infarction (NSTEMI).

For the EP, strain echocardiography may offer a rapid and sensitive tool to determine which patients with NSTEMI would benefit from urgent revascularization. With the advent and implementation of increasingly sensitive troponin assays,^16^ it is likely that there will be an increased proportion of ED patients with abnormal troponin values who do not require urgent revascularization. Currently, the evaluation of these patients with focused echocardiography may be able to identify an obvious wall motion abnormality; however, in the earliest phase of MI there is microvascular obstruction that does not lead to resultant wall motion abnormalities on conventional echocardiography.^17^ The microvascular obstruction results in impaired function of the longitudinally-oriented subendocardial fibers of the LV prior to the development of any overt wall motion changes (Figure 3). Thus, the addition of LV strain to the early assessment of patients with elevated troponins has the potential to identify patients who may benefit from an early and invasive management strategy. A small single center study by Dahlslett et al evaluated patients being evaluated for suspected ACS who had normal initial biomarkers.^18^ They found that a GLS of −21% or better had a sensitivity of 93% to rule out coronary artery disease and was superior to standard echocardiographic parameters used in the evaluation of ischemia. As a comparison exercise echocardiography and dobutamine stress echocardiography have sensitivity ranges of 74–97% (mean 88%) and 61–95% (mean 81%). 19 If the results from this small single center study by Dahlslett can be replicated in larger multi-center trials, strain echocardiography with calculation of GLS has potential to be used in conjunction with other testing strategies in ED patients with suspected ACS.

### Acute Heart Failure

The mechanics of the LV are much more complex than basic 2D imaging is able to depict. As mentioned previously, deformation of the LV occurs in three planes: longitudinal, circumferential, and radial. These vectors of motion are the result of natural myocardial fiber orientation, with subendocardial and subepicardial fibers being arranged in a longitudinal fashion, while mid-wall fibers are arranged in a circular orientation.^20^ In the setting of acute HF, LV dysfunction may exist in any of the different planes, making it challenging to identify abnormalities using conventional methods. Early studies in patients with HF and preserved LVEF found impaired longitudinal strain, with values that were comparable to patients with impaired LVEF.^21^ Given the microscopic anatomy of the LV, the existence of early impairment in longitudinal strain in patients with HF supports a conceptual model where cardiac dysfunction represents more than just preserved or reduced EF. Perhaps more importantly, impaired systolic strain, especially GLS has been shown to be an independent predictor of adverse outcome in patients with HF even when accounting for conventional prognosticators such as LVEF.^22^–^24^

Of particular importance for the ED patient with acute HF, strain is acutely sensitive to the loading conditions of the heart,^21^ whereas the traditional marker of LV systolic function, LVEF, is not. The limits of LVEF as a guide to treatment during the acute phase of HF management were demonstrated in a study by Gandhi et al where they studied 38 patients who presented to the ED with acute hypertensive pulmonary edema.^25^ These patients underwent echocardiography shortly after arrival, while they required respiratory support and vasoactive medications, and again one-three days after the successful treatment of the acute episode. Despite dramatic changes in their hemodynamic profiles (mean initial systolic BP 200+/−26mmHg; follow-up examination mean systolic BP 139+/−17mmHg) as well as significant improvement in dyspnea, the mean LVEF was unchanged (50+/−15% during the acute episode and 50+/−13% after treatment). Eighteen of the patients had a normal LVEF after treatment, 16 of whom also had normal LVEF during treatment.

Although traditional measurements of LV systolic function do not change appreciably during treatment of acute HF, diastolic parameters do demonstrate improvements as patients undergo treatment.^25^,^26^ Diastolic dysfunction is an important mechanism contributing to dyspnea in patients with acute HF irrespective of systolic function.^27^,^28^ The assessment of diastolic function requires standard 2D imaging as well as pulsed Doppler (PW) and tissue Doppler (TDI) performed from the A4C window. Strain assessment of diastolic function of the LV avoids the time-consuming process of obtaining multiple measures at various locations (LV inflow at the mitral valve leaflet tips, septal and lateral annulus of mitral valve and pulmonary veins), and instead it relies on the semi-automated software to measure the amount (strain) and rate (strain rate) of deformation of individual segments of the LV and the LV as a whole.^29^

**Figure 4:**
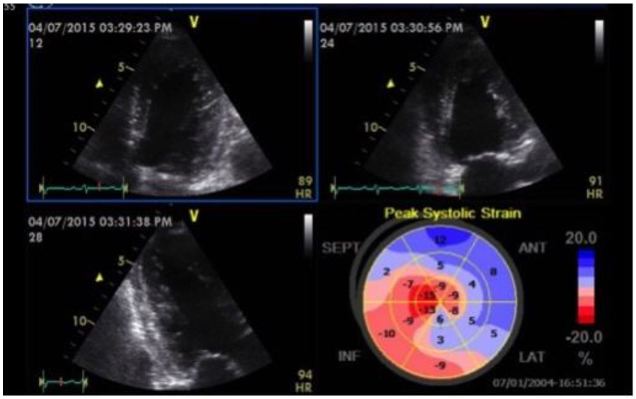
Baseline echocardiogram in a patient with acute hypertensive pulmonary edema. Quad view demonstrating apical 4-chamber, 2-chamber and long axis images with a bullseye map. Global longitudinal strain is −7.1%. There is significant dyskinesis in the anteroseptal, anterior and lateral segments.

**Figure 5:**
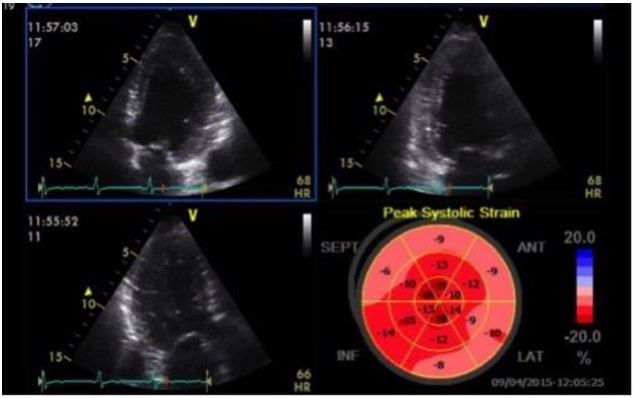
Follow-up echocardiogram of patient from Figure 4 24 hours after presentation. Quad view, Global longitudinal strain is now −13.5%. Anteroseptal, anterior and lateral wall dyskinesis has resolved following afterload reduction.

A recent case from our ED highlights some of these important concepts. A 61-year-old female presented with two days of dyspnea, which had acutely worsened in the last several hours. Her initial blood pressure was 269/181mmHg, heart rate 105 beats per minute, respiratory rate 28 breaths per minute and pulse oximetry 89% on room air. Her lung examination revealed diffuse rates and a focused lung ultrasound showed diffuse B lines consistent with acute cardiogenic pulmonary edema. She was started on non-invasive positive pressure ventilation (NIPPV) and received multiple 2mg boluses of IV nitroglycerin, which were followed by a continuous nitroglycerin infusion. Shortly after the initiation of NIPPV a POC echocardiogram was performed showing mild LV hypokinesis on the standard 2D images, but significantly impaired anteroseptal myocardial strain with a GLS of −7.1% (Figure 4). Approximately two hours after her initial presentation, the patient’s work of breathing had significantly improved so NIPPV was discontinued. Blood pressure was 171/110mmHg at the time. Twenty-four hours after her initial presentation, repeat strain analysis was performed showing a 90% improvement in GLS (−13.5%) with dramatic reversal of anteroseptal dysfunction (Figure 5). As evidenced by this case, assessment of LV strain in acute HF has the potential to identify unique echocardiographic features that can define treatment responsiveness among different clinical phenotypes.

### Right Ventricular Strain

One of the newer applications of strain is the assessment of the right ventricular (RV) free wall.^30^ The ability to use an early, non-invasive method to assess for acute RV dysfunction in situations where submassive pulmonary embolism (PE) is suspected would enable earlier initiation of thrombolytic therapy. RV free wall strain also has the ability to rapidly risk stratify patients who have been diagnosed with PE without requiring time-consuming, technically challenging Doppler techniques. Further, the semi-quantitative assessment with speckle tracking removes much of the subjectivity associated with the determination of RV hypokinesis and the presence of McConnell’s sign. A recent study showed the potential utility of strain echocardiography for this purpose, comparing measures of RV strain in 75 patients with confirmed central PE with 30 control subjects.^31^ Regional and global RV free wall and septal wall strain was assessed offline using speckle-tracking software and both were significantly reduced in PE subjects compared to controls, with no difference based on presence or absence of McConnell’s sign. Additional studies involving patients with acute submassive PE found the combination of mean RV free wall strain <−12%, RV EF <40% on 3D imaging, and RVSP >43mmHg to be present in 94% of those who suffered an adverse event versus 23% of patients without adverse events.^32^ RV EF has not been shown to be a valid reproducible measure of RV systolic function,^33^ and RVSP can be time consuming to determine and also involves using Doppler, thus making RV free wall strain a potentially easy way to calculate singular determination of RV systolic function that would be very useful to the EP caring for critically ill patients with acute PE.

## LIMITATIONS

Strain analysis using speckle-tracking technology does have several limitations that need to be understood prior to the adoption of this as a part of POC echocardiography in the ED. Speckle tracking relies on the ability of the ultrasound system to track specific acoustic markers in the myocardium over time, and is thus dependent on achieving high frame rates. Increasing depth and increasing sector width (among other variables related to the transducer) will decrease the frame rate. Frame rates less than 40 frames per second (Hz), result in large frame-to-frame changes, resulting in poor tracking of acoustic speckles. One strategy we have found useful to improve the frame rate is to decrease depth in the apical windows such that the left atrium is out of the field of view. Another limitation with speckle tracking is found in patients with heart rates >120 beats per minute; as fewer data points from a single cardiac cycle are available, strain data becomes less reliable. Also, if the heart rate is dramatically different from one clip to the next (i.e. 60 beats per minute in the apical long axis and A4C views but 100 beats per minute in the apical wo-chamber) the algorithm will be unable to calculate GLS because of the differences in tracking. As a consequence of beat-to-beat variability, conditions such as atrial fibrillation and multifocal atrial tachycardia are considered a relative contraindication to strain imaging. Fortunately, in our experience, the heart rate and rhythm-related limitations occur infrequently in patients being evaluated for ACS or acute HF.

Beyond technical issues, a lack of standardization of left ventricular (LV) strain between the various ultrasound manufacturers has led to hesitancy in the adoption of strain as a routine measure of cardiac function in some echocardiography labs.^34^ Much research has already been performed comparing various manufactures’ strain algorithms, and leaders in the field of echocardiography have called for manufacturers to work together to standardize strain measurements across vendors.^35^ Lastly, strain analysis does require equipment that is not generally found in most EDs. Many ultrasound manufacturers have equipment that offers speckle-tracking technology; however, this equipment is not typically marketed to the ED and thus many EPs are unfamiliar with it. Also, to our knowledge there is currently no manufacturer that has the ability to upgrade existing equipment by adding software to calculate strain to existing POC ultrasound platforms found in most EDs. Finally, the cost for a machine with speckle-tracking technology is, on average, slightly greater than ultrasound systems commonly used in most EDs (approximately $50,000 for a portable laptop-based system with one matrix array transducer), thus presenting a potential barrier to broad uptake.

## CONCLUSION

Echocardiography in the ED has evolved from a single institution borrowing their cardiology department’s equipment during off-hours to standards of training that have been endorsed by the Accreditation Council for Graduate Medical Education and adopted by all emergency medicine residencies. A core curriculum currently exists for focused echocardiography in the ED with assessment of the pericardium, LV systolic function, the right heart, preload determination, and procedural guidance. We believe that the next step in the development of the field is to adopt novel approaches such as strain echocardiography using speckle-tracking software to assist in the management of patients with HF, MI and PE. The advent of portable ultrasound systems and semi-automated software to calculate strain makes this a real possibility for ED POC echocardiography in the near-term. Once EPs gain familiarity with the speckle-tracking software, a research agenda will need to follow, with a goal of studying the utility of strain imaging at the POC. The net result will be a more individualized, image-based understanding of conditions such as ACS, acute HF, and PE with the potential to develop treatment protocols directed at echocardiographic phenotypes.

## Figures and Tables

**Table:** Normal left ventricular strain values.

Study	# subjects	Mean age (yrs)	Manufacturer	Technique	Mean global longitudinal strain	
Marwick et al. 2009^1^	192	51+/−12	GE Vivid 7	Speckle tracking		−18.6%+/−0.1%
Nakai et al. 2009^2^	25	62+/−11	GE Vivid 7	Speckle tracking		−20.8%+/−1.8%
Manovel et al. 2010^3^	28	38+/−12	GE vs Toshiba	Speckle tracking	GE:	−21.95%+/−1.8%
					Toshiba:	−22.28%+/−2.1%
Biaggi et al. 2011^4^	47	37+/−10	GE Vivid 7	Speckle tracking (GE vs Siemans software)	GE:	−21.9%+/−2.0%
					Toshiba:	−20.9%+/−2.4%
Takigiku (JUSTICE) 2012^5^	817	36+/−18	GE vs Phillips vs Toshiba	Speckle tracking	GE:	−21.3%+/−2.1%
					Phillips:	−18.9%+/−2.5%
					Toshiba:	−19.9%+/−2.4%
Sun et al. 2013^6^	228	44+/−15	Phillips	Speckle tracking		−20.4%+/−3.4%

1 Marwick TH, Leano RL, Brown J, et al. Myocardial Strain Measurement with 2-Dimensional Speckle-Tracking Echocardiography. Definition of Normal Range. *JACC Cardiovasc Imaging*. 2009;2(2):80–4.

2 Nakai H, Takeuchi M, Nishikage T, et al. Subclinical Left Ventricular Dysfunction in Asymptomatic Diabetic Patients Assessed by Two-Dimensional Speckle Tracking Echocardiography: Correlation with Diabetic Duration. *Eur J Echocardiog*. 2009;10(8):926–932.

3 Manovel A, Dawson D, Smith B, et al. Assessment of Left Ventricular Function by Different Speckle-Tracking Software. *Eur J Echocardiog*. 2010;11(5):417–421.

4 Biaggi P, Carasso S, Garceau P, et al. Comparison of Two Different Speckle Tracking Software Systems: Does the Method Matter? *Echocardiography*. 2011;28(5):539–547.

5 Takigiku K, Takeuchi M, Izumi C, et al. Normal Range of Left Ventricular 2-Dimensional Strain. Japanese Ultrasound Speckle Tracking of the Left Ventricle Study. *Circ J*. 2012;76(11):2623–32.

6 Sun JP, Pui-Wai Lee A, Wu C, et al. Quantification of Left Ventricular Regional Myocardial Function Using Two–Dimensional Speckle Tracking Echocardiography in Healthy Volunteers- A Multi-Center Study. *Int J Cardiol*. 2013;167(2):495–501.
